# Spry1 and Spry4 Differentially Regulate Human Aortic Smooth Muscle Cell Phenotype via Akt/FoxO/Myocardin Signaling

**DOI:** 10.1371/journal.pone.0058746

**Published:** 2013-03-15

**Authors:** Xuehui Yang, Yan Gong, Yuefeng Tang, Hongfang Li, Qing He, Lindsey Gower, Lucy Liaw, Robert E. Friesel

**Affiliations:** 1 Center for Molecular Medicine, Maine Medical Center Research Institute, Scarborough, Maine, United States of America; 2 Graduate School for Biomedical Sciences, University of Maine, Orono, Maine, United States of America; 3 Department of Physiology, College of Basic Medicine, Lanzhou University, Lanzhou, China; University of Sassari, Italy

## Abstract

**Background:**

Changes in the vascular smooth muscle cell (VSMC) contractile phenotype occur in pathological states such as restenosis and atherosclerosis. Multiple cytokines, signaling through receptor tyrosine kinases (RTK) and PI3K/Akt and MAPK/ERK pathways, regulate these phenotypic transitions. The Spry proteins are feedback modulators of RTK signaling, but their specific roles in VSMC have not been established.

**Methodology/Principal Findings:**

Here, we report for the first time that Spry1, but not Spry4, is required for maintaining the differentiated state of human VSMC *in vitro*. While Spry1 is a known MAPK/ERK inhibitor in many cell types, we found that Spry1 has little effect on MAPK/ERK signaling but increases and maintains Akt activation in VSMC. Sustained Akt signaling is required for VSMC marker expression in vitro, while ERK signaling negatively modulates Akt activation and VSMC marker gene expression. Spry4, which antagonizes both MAPK/ERK and Akt signaling, suppresses VSMC differentiation marker gene expression. We show using siRNA knockdown and ChIP assays that FoxO3a, a downstream target of PI3K/Akt signaling, represses myocardin promoter activity, and that Spry1 increases, while Spry4 decreases myocardin mRNA levels.

**Conclusions:**

Together, these data indicate that Spry1 and Spry4 have opposing roles in VSMC phenotypic modulation, and Spry1 maintains the VSMC differentiation phenotype in vitro in part through an Akt/FoxO/myocardin pathway.

## Introduction

Phenotypic modulation of vascular smooth muscle cells (VSMC) plays a critical role in the development of vascular diseases such as atherosclerosis and post-angioplasty restenosis [1], [2]. VSMC in the adult vessel exhibit a fully differentiated phenotype with low proliferation and high expression of smooth muscle markers including SM alpha-actin (ACTA2), SM22 alpha (SM22α), calponin-h1 (CNN1), SM myosin heavy chain (MYH11), and smoothelin B (SMTN-B) [1], [2]. However, VSMC in the intact vessel wall retain phenotypic plasticity, and can respond to environment changes, such as injury, by loss of expression of contractile genes, hyperproliferation, and migration to form a neointima. Elucidation of the mechanisms of VSMC phenotypic switching is critically important to provide insight towards a better understanding of the development of vascular disease and its treatment.

Cellular signaling pathways including phosphatidylinositol 3-kinase/protein kinase B (PI3K/Akt), extracellular signal-regulated kinase (MAPK/ERK) and p38 mitogen-activated protein kinase (p38) regulate VSMC phenotypic modulation [3], [4], [5], [6], [7]. The PI3K/Akt signaling pathway promotes both proliferation and, paradoxically, differentiation of VSMC, while MAPK/ERK signaling mediates proliferation and migration of VSMC. The expression of VSMC marker genes is dependent upon a cis-acting DNA sequence, the CArG box, which binds serum response factor (SRF). Myocardin (Myocd) is a potent transcriptional co-activator of SRF, forms an SRF/Myocd transcriptional complex and drives CArG box-dependent VSMC marker gene expression [8]. Elk1, a downstream target of ERK, is a potent repressor of the VSMC contractile gene transcription program by binding to SRF and inhibiting complex formation with Myocd [9]. Phenotypic modulation of VSMC by activation of the Akt pathway occurs in part through FoxO transcription factors. Phosphorylation of FoxOs by Akt leads to their exclusion from the nucleus and inhibiting their transcriptional functions. When Akt signaling is low in VSMC, nuclear FoxO4 forms a complex with Myocd and inhibits transcription of CArG box-dependent genes [10]. Activation of the Akt pathway in VSMC results in dissociation of FoxO4-Myocd complexes and expression of VSMC marker genes.

Spry proteins are feedback regulators of receptor tyrosine kinases (RTKs) that restrain RTK-mediated ERK signaling [11], [12], and therefore play critical roles in the regulation of cell proliferation, differentiation, and survival. By inhibiting ERK signaling, Sprys regulate cell proliferation and differentiation in many cell types including endothelial [13], [14], C2C12 [15], and skeletal muscle satellite cells [16]. Regulation of Akt activation by Sprys is less well understood. One report showed that human Spry2 expression in HeLa cells increases PTEN expression, decreases its phosphorylation and increases its phosphatase activity, leading to decreased Akt activation [17]. However, potential regulation of Akt by Spry in non-transformed cells has not been addressed. In addition, although a TAT-hSpry2 fusion protein was previously reported to inhibit rat VSMC proliferation and migration *in vitro*, and injury induced neointima formation *in vivo* [18], Spry1 and Spry4, which are both highly expressed in normal blood vessels, have not been studied in VSMC. Because of the importance of ERK and Akt signaling in VSMC phenotypic modulation, we addressed the role of Sprys in determining VSMC phenotype. In this study, we report for the first time that Spry1 and Spry4 have opposing effects on Akt activation. Spry1 induces and maintains Akt activation and subsequent expression of VSMC marker genes, while Spry4 inhibits Akt activation and inhibits VSMC proliferation, migration, and VSMC marker gene expression. Using siRNA knockdown and ChIP assays, we demonstrate that FoxO3a binds to the *Myocd* promoter and negatively regulates *Myocd* expression. In consistent, we show wild type FoxO3a and an Akt phosphorylation sites mutant FoxO3aTM inhibit mouse *Myocd* promoter luciferase activity. In addition, we show that Spry1 increases, while Spry4 decreases *Myocd* mRNA expression. Together these data indicate that Spry1 and Spry4 have opposing roles in regulating VSMC phenotype, and Spry1 maintains VSMC differentiation in vitro at least in part through an Akt/FoxO/Myocd pathway.

## Experimental Procedures

### Cell Culture and Manipulation

Human aortic smooth muscle cells (hAoSMC) from at least three individual donors were obtained from Lonza and Invitrogen and cultured in SmGM-2 complete medium (Lonza). hAoSMC were used from passage 4 to 6 for all experiments. For overexpression studies, hAoSMC were plated in 6-well plates at subconfluent density, and transduced with LacZ, Myc-tagged mouse Spry1 or Spry4 adenoviruses at a concentration of 400 virus particles per cell. After overnight incubation with virus, medium was replaced with fresh SmGM-2 and cells were incubated for an additional 24 to 48 h. For knockdown studies, hAoSMC were transduced with human Spry1 or Spry4 shRNA lentiviruses (Open Biosystems) and selected with 1 µg/ml puromycin for 48 h. Transduced cells were incubated for another 48 h in SmGM-2 medium without puromycin. For examining the signaling pathways in regulation of SMC differentiation, hAoSMC were treated with 10 µM U0126 (Cell Signaling) or 10 µM Ly294002 (Cell Signaling) in SmGM-2 medium.

### Western Blot and Antibodies

Cells were lysed in HNTG (50 mM HEPES, pH 7.4, 150 mM NaCl, 1% Triton X-100, 5 mM EGTA) buffer containing phosphatase inhibitors (1 mM sodium orthovanadate and 1 mM NaF) and a proteinase inhibitor cocktail (Roche). Cell lysates were subjected to immunoblotting using antibodies to ACTA2 (SMA, Sigma) (1∶5000), SM22α (Abcam) (1∶2000), calponin (Abcam) (1∶1000), SMTN-B (Santa Cruz, 1∶1000), cyclin D1 (1∶1000), phospho-Akt (S473) and Akt (1∶1000), phospho-FoxO1/FoxO3a, phospo-FoxO4, FoxO1, FoxO3a and FoxO4 (Cell Signaling, 1∶1000), phospho-ERK (Sigma, 1∶10000), ERK1/2, and Myc (Santa Cruz, 1∶1000), beta-actin or tubulin (Sigma, 1∶5000).

### RT-PCR and Quantitative Real-time PCR

Total RNA was extracted from hAoSMC using RNeasy Plus (Qiagen). The purity and concentration of total RNA were measured with NanoDrop Spectrophotometer (NanoDrop Technologies) at 260 nm/280 nm. The ratios of 260 nm/280 nm of all samples were between 1.8 and 2.0. ProtoScript M-MuLA First Strand cDNA Synthesis kit (Biolab) was used to generate cDNA. Quantitative real-time PCR (qPCR) of target genes was performed using SYBR Green (SABiosciences) on an IQ5 Multicolor Real-Time PCR Detection System (BioRad) according to the manufacturer’s instructions. GAPDH was used as an internal reference in each reaction. Melting curve analyses using the program run in the step acquisition mode was used to verify the presence of a single amplification production. Primers for qPCR are shown in Table S1.

### Immunostaining and FACS Analysis

All procedures involving human samples were approved by the Maine Medical Center Institutional Review Board (IRB), and conducted in compliance with ethical and safe research practices involving human subjects. Paraffin embedded specimens from surgically resected arteries were sectioned at 5 µM and stained with Spry1, Spry2 or Spry4 antibodies (Santa Cruz) followed by color development using DAB peroxidase substrate (Vector Laboratories). The Maine Medical Center Institutional Animal Care and Use Committee approved all procedures involving animals. Mouse carotid arteries were fixed in 10% formalin, embedded in OCT, sectioned at 5 µM and co-stained with Cy3-conjugated SMA antibodies and Spry1, Spry2 or Spry4 antibodies followed by FITC-anti-rabbit antibody. For in vitro cell immunostaining, hAoSMC were transduced with Spry1, Spry4 adenoviruses and shRNA lentiviruses. For Ki67 immunostaining analysis, transduced cells were collected by trypsin digestion and fixed in 4% paraformaldehyde (PFA) for 10 min, stained with FITC-Ki67 antibody (Santa Cruz, 1∶50). Fluorescent activated cell sorting (FACSCalibur, BD) was used to analyze the number of Ki67 positive cells. For phospho-histone3 (pH3) and FoxO3a immunostaining, cells were fixed in 4% PFA for 10 min, stained with anti-pH3 (Upstate, 1∶200) or anti-FoxO3a (Cell Signaling, 1∶50) followed by FITC-anti-rabbit antibody (BioRad). Nuclei were counter stained with DAPI, and pH3 positive cells were quantified. Images were acquired using a Leica DMIRB microscope.

### Migration Analysis

hAoSMC were plated in 6 well plates, and triplicate cultures were transduced with Spry1, Spry4 or LacZ adenoviruses overnight. Cells were cultured in fresh complete medium for another 24 h and treated with 2 µg/ml mitomycin C for 2 h prior to wound healing assay. Forty-eight h later, cells were fixed in 4% PFA, stained with Cy3-anti-Myc (Sigma, 1∶200) and nuclei counterstained with DAPI. Images were obtained using a Leica DMIRB fluorescence microscope.

### Luciferase Reporter Assays

293T cells were cultured in DMEM contain 10% FBS. Cells were plated into 12-well plates and co-transfected with Renilla luciferase control (RL-SV40), rat *ACTA2* promoter luciferase (rACTA2-P, gift from Dr.Gary Owens), forkhead response element luciferase (FHRE-luciferase, Addgene), or mouse *Myocd* promoter-luciferase [19] (gift from Dr. Joseph Miano). Each transfection was carried out in triplicate. Luciferase activities were measured with the Dual-Luciferase reporter system (Promega) according to manufacturer’s instructions, and are presented as relative ratio to Renilla luciferase activity.

### Chromatin Immunoprecipitation (ChIP) Analysis

hAoSMC were plated into 10 cm dishes and cultured in SmGM-2 medium with or without U0126 or Ly294002 (10 µM) for 48 h. Cells were cross -linked with 4% formaldehyde for 10 min at 37°C. Cross-linked cells were lysed and subjected for ChIP assay with FoxO3a antibody (Bethyl Laboratory Inc.) using ChIP kit (Millipore) according to manufacturer’s instruction. The primers were designed to span each putative FoxO binding site in the human *Myocd* promoter (GenBank NG_012972). Primer #1 forward is: 5′-tgattttgaccccctgagac-3′, reverse is: 5′-taggctgatttcgtgtgcag-3′; primer #2 forward is: 5′-aagggaacatgGatggattg-3′, reverse is: 5′-tagctcatggggacaacctc-3′; primer #3 forward is: 5′-tttttatgttctgagccaccaa-3′, reverse is: 5′-cctgcgaatgagtctctcct-3′.

### Statistics

Immunoblot and RT-qPCR results are expressed as means of at least three independent experiments. Error bars represent the standard deviation. Comparisons between two groups were performed by Student’s *t* test. For multiple comparisons, Student’s *t* test in conjunction with ANOVA analysis was carried out. *P* values ≤0.05 were considered statistically significant.

## Results

### Spry1 but not Spry4 is Necessary to Maintain Smooth Muscle Marker Gene Expression in hAoSMC

**Figure 1:**
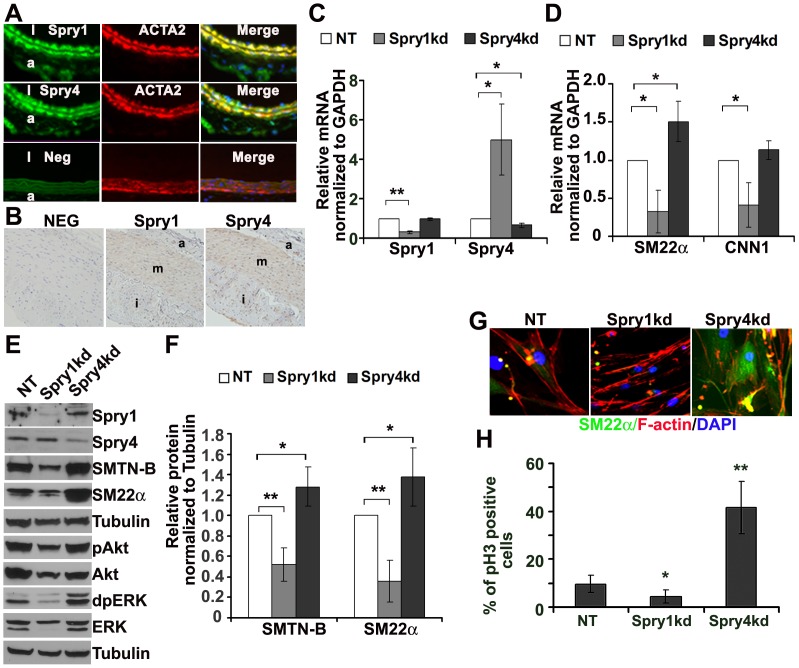
Spry1 is essential to maintain hAoSMC marker gene expression. A) Co-immunofluorescence staining of Spry1, Spry4 and ACTA2 on mouse artery sections show that both Spry1 and Spry4 are expressed in normal arterial smooth muscle cells. Images are from original 400× magnification. B) Immunohistochemistry of Spry1 and Spry4 on human atherosclerotic artery samples showed the expression of Spry1 and Spry4 in artery media smooth muscle cells, and a decreased expression of Spry1 and Spry4 in neointima cells. Images are from original 200× magnification. C) Quantification of RT-qPCR from three independent experiments shows efficacy of knockdown (kd) of Spry1 and Spry4 mRNA by their respective shRNAs compared to a non-targeting sequence (NT). D) Quantification of RT-qPCR from three independent experiments shows that knockdown of Spry1 decreases, whereas knockdown of Spry4 increase VSMC differentiation gene expression at the mRNA level. E) Representative immunoblot showing that knockdown of Spry1 results in a decrease in total Akt and ERK proteins levels, as well as pAkt and pERK. The expression of VSMC marker genes SMTN-B and SM22α is decreased by knockdown of Spry1, whereas knockdown of Spry4 increases pAkt and pERK levels as well as SMTN-B and SM22α. F) Quantification of VSMC marker protein levels from at least three independent immunoblotting experiments. G) Immunofluorescence staining shows down-regulation of SM22α following knockdown of Spry1. Images are from original 400× magnification. H) Quantification of phospho-histone 3 (pH3) immunofluorescence staining from triplicate experiments shows that knockdown of Spry1 reduces pH3 staining, while knockdown of Spry4 increases pH3 staining. Note: a: adventia, m: media, i: intima l: lumen; **: p<0.01, *: p<0.05.

RTK-mediated signaling pathways play critical roles in modulating VSMC phenotype. Immunofluorescence staining shows that Spry1, Spry2 and Spry4 are expressed in ACTA2 expressing smooth muscle cells of the mouse arterial wall (Fig. 1A, and data not shown). We also observed expression of Spry1 and Spry4 in archival samples of human atherosclerotic plaques, with decreased expression of Spry1 and Spry4 observed in neointima cells (Fig. 1B). To determine the role of endogenous Spry1 and Spry4 in VSMC phenotypic modulation, we use lentiviral delivered shRNAs to knockdown Spry1 and Spry4. This approach led to efficient suppression of Spry1 and Spry4 transcripts (Fig. 1B) and protein (Fig. 1D) (the reduction of mRNA of Spry1>90%, p<0.01, and of Spry4 ∼50%, p<0.05). Interestingly, we saw a consistent upregulation of Spry4 mRNA and protein in hAoSMC when Spry1 was silenced. More importantly, knockdown of Spry1 significantly decreased VSMC marker gene expression as determined by immunoblotting, RT-qPCR, and immunofluorescence microscopy, while knockdown of Spry4 increased VSMC marker gene expression compared to non-targeting control shRNA (Fig. 1C–F).

Because the activation of Akt and ERK are important in regulating VSMC phenotype, we performed immunoblotting to examine whether knockdown of Spry1 or Spry4 affects cellular signaling pathways. Knockdown of Spry4 in hAoSMC increased ERK and Akt phosphorylation (Fig. 1D), however, surprisingly, knockdown of Spry1 consistently decreased phosphorylated and total levels of ERK and Akt. We also observed an impairment of cell growth in Spry1 shRNA-treated hAoSMC, while knockdown of Spry4 increased cell proliferation as determined by phospho-histone 3 (pH3) immunostaining (Fig. 1G). To validate these results, we also used siRNA Smartpools® (Dharmacon) to knockdown Spry1 or Spry4 and obtained similar results (data not shown). These data indicate that Spry1 and Spry4 have opposing effects on cell proliferation and differentiation of VSMC, and that Spry1 is essential to maintain hAoSMC marker gene expression.

### Spry1 Promotes hAoSMC Differentiation by Increasing the PI3K/Akt Signaling Pathway

**Figure 2:**
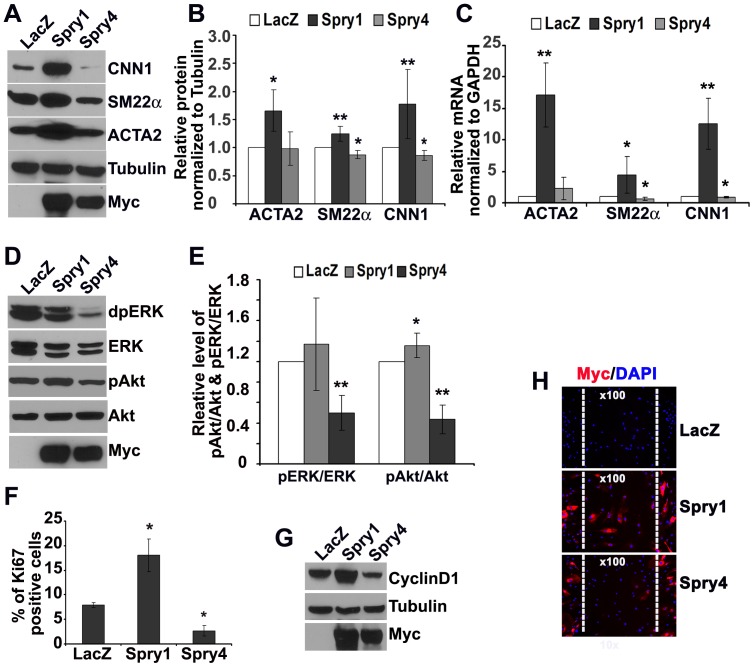
Spry1 and Spry4 differently regulate Akt activation, hAoSMC differentiation and proliferation. A) Representative immunoblot shows that overexpression of Spry1 increases VSMC marker CNN1, ACTA2 and SM22α expression, whereas overexpression of Spry4 decreases expression of these markers. B) Quantification of VSMC marker protein expression from at least three independent immunoblotting experiments show that overexpression of Spry1 significantly increases ACTA2, CNN1 and SM22α, whereas overexpression of Spry4 has small but statistically significant decrease of CNN1 and SM22α. C) Quantification of RT-qPCR from three independent experiments shows that overexpression of Spry1 increases VSMC marker gene mRNA (*SM22α*, and *CNN1*), whereas over expression of Spry4 decreases these gene mRNA expression. D) Representative immunoblot to show that Spry1 has little effect on pERK but increases pAkt, while Spry4 decreases both pERK and pAkt in hAoSMC. E) Quantification of the relative levels of pERK vs. ERK and pAkt vs. Akt from at least three independent immunoblotting experiments. F) Flow cytometric analysis shows that Spry1 increases, while Spry4 decreases hAoSMC proliferation as indicated by the percentage of Ki67 positive cells. Quantification is from three independent experiments. G) Representative immunoblot shows that Spry1 increases cyclinD1 expression. H) Cell migration wound healing assay. Representative images show that more Myc-positive Spry1 overexpressing cells migrated into wound area compared to Myc-positive Spry4 overexpressing cells 48 hours after wounding. Mitomycin treatment was used to eliminate the effect of cell proliferation. Note: *: p<0.05; **: p<0.01.

To further evaluate the role of Spry1 and Spry4 in VSMC, we overexpressed myc-tagged mouse Spry1 or Spry4 in hAoSMC using an adenoviral (Ad) expression system. In contrast to knockdown of Spry1 or Spry4, overexpression of Spry1 increased VSMC markers expression such as ACTA2, CNN1and SM22α both at the protein and mRNA levels (Fig. 2A–C). Conversely, adenovirus-mediated expression of Spry4 (AdSpry4) decreased expression of CNN1 and SM22α, with little effect on ACTA2 expression (Fig. 2A–C). Expression of Spry4 significantly inhibited ERK and Akt phosphorylation, whereas expression of Spry1 had little effect on ERK phosphorylation (Fig 2D, E). In contrast, Spry1 increased Akt phosphorylation in hAoSMC (Fig. 2D, E). Spry4 overexpression also inhibited cell proliferation as determined by Ki67 immunostaining and fluorescence activated cell-sorting analysis (Fig. 2F). Surprisingly, overexpression of Spry1 resulted in increased cell proliferation relative to control. Immunoblotting revealed that over-expression of Spry1 increased cyclin D1 protein, whereas Spry4 mildly decreased cyclin D1, consistent with an increase in cell proliferation by Spry1 and a decrease in cell proliferation by Spry4 (Fig. 2G).

PI3K/Akt and MAPK/ERK signals play a critical role in regulating VSMC migration. It is important to determine whether Spry1 and Spry4 have effects on hAoSMC migratory behavior. AdSpry1, AdSpry4 or AdLacZ transduced hAoSMC were pre-treated with 2ug/ml mitomycin to eliminate the effect of cell proliferation on wound healing. The efficacy of AdSpry1 and AdSpry4 transduction was about 75% based on the anti-Myc immunofluorescence analysis (data not shown). Figure 2H shows that 48 hours after wounding the total cells migrated into wound areas was similar among AdLacZ, AdSpry1 and AdSpry4 manipulated groups, however, ∼90% of cells in wound area of AdSpry1 group are Myc-positive, while only ∼10% of cells in wound area of AdSpry4 group are Myc-positive. These results suggest that Spry1 weakly promotes hAoSMC migration, while Spry4 inhibits hAoSMC migration.

**Figure 3:**
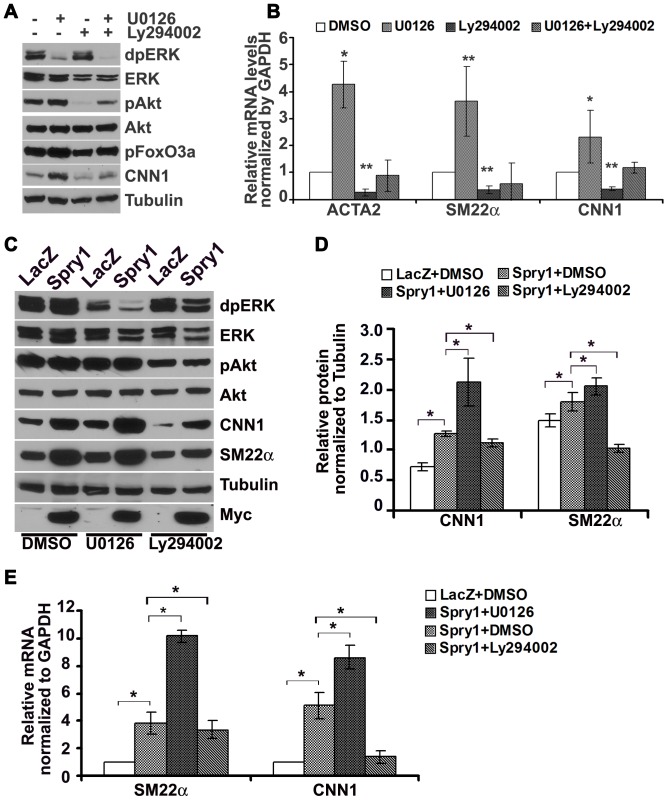
Activation of PI3K/Akt is essential for Spry1 mediated VSMC marker induction. A) Representative immunoblot shows that inhibition of PI3K by Ly294002 decreases CNN1 expression, while inhibition of MEK by U0126 increases pAkt and CNN1 expression. Combined treatment with Ly294002 and U0126 partly reverses Ly294002 mediated Akt activation and CNN1 expression. B) Quantification of three independent immunoblotting experiments to show that sustained PI3K/Akt signaling is necessary for VSMC markers expression in hAoSMC in vitro, whereas MAPK/ERK signaling negatively regulates Akt activation and its induction of VSMC markers expression. C) Representative immunoblot shows that inhibition of PI3K/Akt signaling by Ly294002 abolishes Spry1 induced VSMC markers expression. D) Quantification of at least three independent immunoblotting experiments to show maintaining and inducing PI3K/Akt signaling is responsible to Spry1 medicated up-regulation of VSMC markers expression. E) Quantification of RT-qPCR from three independent experiments to confirm that inhibition of PI3K/Akt by Ly294002 abolished Spry1 induced SMC marker mRNA expression. Note: *: p<0.05; **: p<0.01.

We used chemical inhibitors of the ERK and Akt pathways to examine the impact of these signaling pathways on VSMC marker gene expression in hAoSMC grown in complete medium. Treatment of hAoSMC with the MEK inhibitor U0126 significantly inhibited ERK activation, but surprisingly increased pAkt and pFoxO3a, with a corresponding increase in the expression of VSMC marker genes compared to vehicle control (Fig. 3A-B). The PI3K inhibitor Ly294002 inhibited phosphorylation of Akt and FoxO3a and decreased VSMC markers protein and mRNA expression compared to vehicle control (Fig. 3A–B). Finally, in co-treatment setting U0126 partially restored Ly294002 mediated down-regulation of Akt and FoxO3a phosphorylation, as well as VSMC markers gene expression. Together these data suggest that Akt signaling plays a major role in VSMC phenotypic modulation, activation of Akt promotes VSMC markers expression while ERK signaling negatively affects Akt signaling in this context. Because Spry1 increased VSMC marker gene expression accompanied by an increase of pAkt, we treated AdSpry1-transduced hAoSMC with U0126 and Ly294002 to gain additional insight into the pathways involved. Immunoblotting revealed that the PI3K inhibitor Ly294002 decreased Akt phosphorylation in Spry1 over-expressing hAoSMC, and reduced the induction of SM22α and CNN1 by Spry1 (Fig. 3C–D). However, the MEK inhibitor U0126 further increased Spry1-mediated induction of SM22α and CNN1 protein levels, consistent with a role for ERK as negative regulator of Akt signaling and VSMC marker gene expression (Fig. 3C–D). RT-qPCR analysis confirmed that inhibition of PI3K/Akt signaling with Ly294002 abrogated AdSpry1 mediated up-regulation of SM22α and CNN1, while inhibition of MAPK/ERK signaling further increased Spry1 mediated expression of these markers (Fig. 3E). Thus, activation of PI3K/Akt signal is required for Spry1-mediated up-regulation of VSMC markers.

### FoxO3a Represses Myocd Expression and Spry1 Promotes VSMC Marker Gene Expression in Part through Activating Akt and Relieving the Repressive Function of FoxO on Myocd Transcription

Because the increase in Akt activity induced by Spry1 overexpression contributed to the induction of hAoSMC marker expression, we sought to further study the role of Akt signaling in this process. Akt phosphorylates FoxO proteins, thus abrogating their activity. FoxO4, but not FoxO1 or FoxO3a, inhibits VSMC marker gene expression by binding to and inhibiting the transcriptional activity of the SRF/Myocd complex [10]. We examined expression of FoxO family members in hAoSMC, and found abundant FoxO3a expression and lower levels of FoxO1 and FoxO4 (Fig. S1). In addition, we confirmed the previous report that forced expression of FoxO4, but not FoxO3a inhibits ectopic expression of Myocd mediated expression of CNN1 and rACTA2-P luciferase activity in 293T cells (Fig. S1) [10].

**Figure 4:**
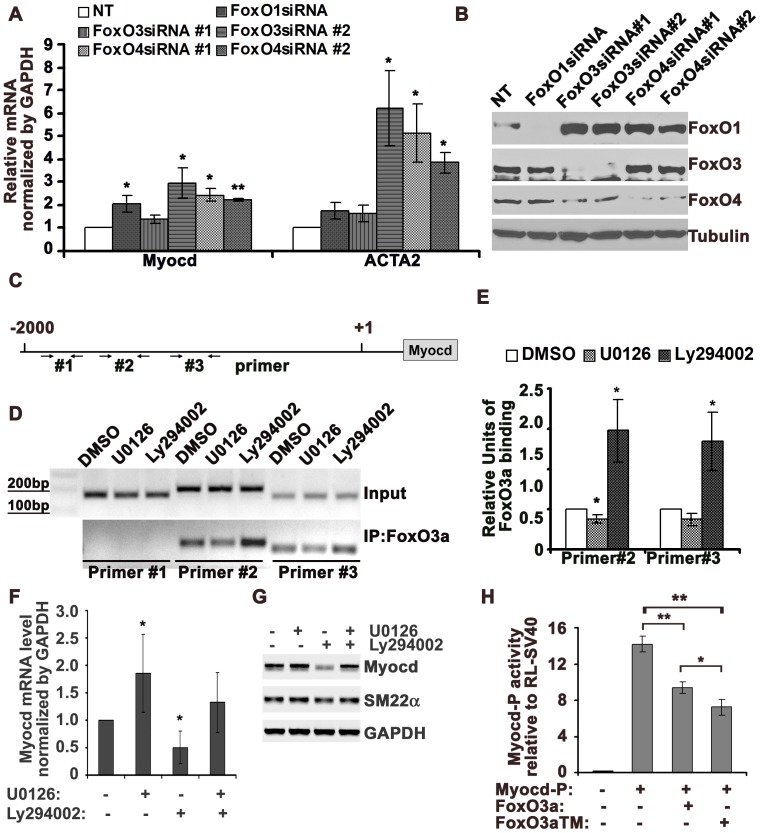
FoxO3a proteins bind *Myocd* promoter and negatively regulate *Myocd* and VSMC marker expression, Akt activation releases its repressive effect. A) Quantification of RT-qPCR from three independent experiments shows that knockdown of *FoxO1*, *FoxO3a* and *FoxO4* by respective siRNAs increases *Myocd* and *ACTA2* mRNA levels. B) Representative immunoblot shows an effective knockdown of FoxO3a and FoxO4 by their respective siRNAs. C) Schematic of primer design that span each putative FoxO binding site on the *Myocd* promoter for ChIP analysis. D) Representative anti-FoxO3a ChIP-PCR showing that U0126 inhibits, whereas Ly294002 promotes FoxO3a binding to the *Myocd* promoter. E) Quantification of qPCR from three independent analyses of anti-FoxO3a ChIP assays. F) Quantification of RT-qPCR from three independent experiments shows that inhibition of MEK signaling by U0126 increases *Myocd* mRNA expression, while inhibition of PI3K activation by Ly294002 decreases *Myocd* mRNA expression, while combined treatment with the two inhibitors partially reverses the decrease of *Myocd* mRNA expression by Ly294002. G) Agarose gel analysis shows that the PI3K inhibitor Ly294002 down-regulates *Myocd* transcription. H) Promoter luciferase assay shows that wild type FoxO3 and triple mutant of Akt phosphorylation sites FoxO3aTM significantly decreases mouse *Myocd*-P luciferase activities. These experiments were performed three times in triplicate. Note: *: p<0.05; **: p<0.01.

It was reported that FoxO3a negatively regulates Myocd expression in cardiomyocytes through up-regulating catalase and subsequently reducing reactive oxygen species [20]. We sought to determine whether FoxO proteins regulate Myocd expression in hAoSMC. We knocked down FoxO1, FoxO3a and FoxO4 using siRNA, and examined the expression of *Myocd* and VSMC marker *ACTA2* whose promoter contains CArG-box that binds to SRF/Myocd and regulated by Myocd. RT-qPCR showed that knockdown of FoxO1, FoxO3a or FoxO4 increased *Myocd* mRNA and *ACTA2* expression (Fig. 4A). Immunoblotting showed an effective knockdown either FoxO3 or FoxO4 (Fig. 4B). By *in silico* analysis of the human *Myocd* promoter (GenBank NG_012972), we found three putative FoxO binding sites: TGTTTTA located at position −1791 and −1261 and TGTTTTC located at position −1533 [21] (Fig. 4C). Because knockdown of FoxOs resulted in increased *Myocd* expression, we hypothesize FoxOs repress *Myocd* transcription by binding to the *Myocd* promoter. Due to the abundance of the FoxO3a isoform in hAoSMC, we performed chromatin immunoprecipitation (ChIP) assays using FoxO3a antibodies to determine whether FoxO3a interacts with the *Myocd* promoter. ChIP-PCR showed that FoxO3a interacts with two of the three putative FoxO binding sites of the *Myocd* promoter, and treatment of hAoSMC with the PI3K inhibitor Ly294002 resulted in an increase in FoxO3a interaction with the *Myocd* promoter compared to control cells or cells treated with the MEK inhibitor U0126 (Fig. 4D–E). In addition, PI3K inhibitor Ly294002 treatment significantly decreased *Myocd* transcripts in hAoSMC (Fig. 4F–G). The MEK inhibitor U0126, which increased Akt activation (Fig. 3A), also upregulated *Myocd* expression, whereas combined treatment with U0126 and Ly294002 partially reversed Ly294002 mediated *Myocd* down-regulation. Further more, promoter luciferase assay showed that FoxO3a decreased *Myocd* promoter luciferase activity, while FoxO3aTM, a mutant deficient in Akt phosphorylation sites further decreased *Myocd* promoter luciferase activity (Fig. 4H). These data suggest that an additional mechanism by which FoxO proteins, especially FoxO3a, regulate VSMC marker gene expression by repressing *Myocd* expression in hAoSMC.

**Figure 5:**
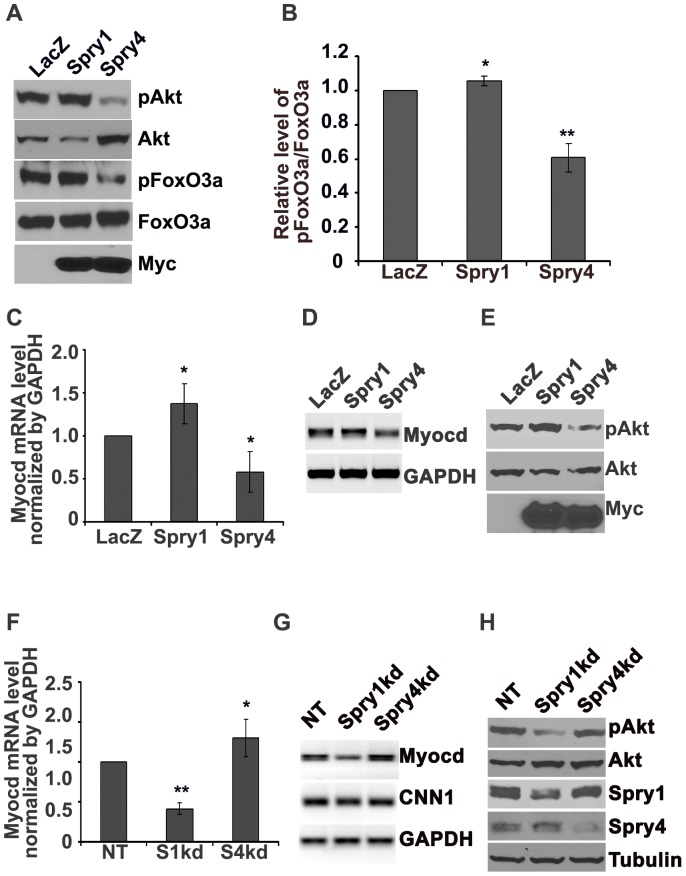
Spry1 increases, Spry4 decreases *Myocd* expression associated with their corresponding regulation of PI3K/Akt signaling. A) Representative immunoblot to verify overexpression of Spry1 increases, while overexpression of Spry4 decreases pAkt and pFoxO3a. B) Quantification from at least three independent immunoblotting experiments shows Spry1 increases, while Spry4 decreases pAkt and pFoxO3a levels. C) Quantification of RT-qPCR from three independent experiments showing that overexpression of Spry1 increases, while overexpression of Spry4 decreases *Myocd* mRNA expression. D) Representative agarose gel analysis shows Spry1 increases, Spry4 decreases *Myocd* mRNA expression. E) Parallel experiment to C and D for immunoblotting assay to confirm overexpression of Spry1 increased pAkt, while overexpression of Spry4 decreased pAkt. F) Quantification of RT-qPCR from three independent experiments showing that knockdown of Spry1 decreases, while knockdown of Spry4 increases *Myocd* mRNA expression. G) Representative agarose gel analysis shows knockdown of Spry1 decreases, while knockdown of Spry4 increases *Myocd* mRNA expression. H) Parallel experiment to F and G for immunoblotting assay to verify knockdown of Spry1 decreased pAkt, while knockdown of Spry4 increased pAkt. Note: *: p<0.05, **: p<0.01.

Using immunoblot analysis we verified that overexpression of Spry1 increased pAkt as well as pFoxO3a levels, while forced expression of Spry4 decreased pAkt and pFoxO3a levels (Fig. 5A–B), and increased FoxO3a nuclear localization (Fig. S2). In addition, Spry1 overexpression in 293T cells decreased fork head response element luciferase (FHRE-luciferase) activity, further confirming the role of Spry1 in the modulation of Akt/FoxO signaling. However, because of low basal Akt activity in these cells after 48h in serum containing medium, overexpression of Spry4 did not further increase FHRE-Luciferase activity relative to control (Fig. S2). By RT-qPCR analysis we observed that overexpression of Spry1 resulted in an increase in *Myocd* expression along with an increase of Akt activation, whereas forced expression of Spry4 resulted in decreased expression of *Myocd* and decreased pAkt (Fig. 5C–E) in hAoSMC. In contrast, knockdown of Spry1 decreased *Myocd* expression and Akt activation, while knockdown of Spry4 increased *Myocd* expression and Akt activation (Fig. 5F–H). Together, these data indicate that Spry1, by positively influencing Akt activation, induces the expression of *Myocd* in part through a mechanism of releasing the repression of FoxO3a on *Myocd* promoter activity, while Spry4 decreases *Myocd* expression by inhibiting Akt activation and increasing FoxO3 nuclear translocation and repression on *Myocd* promoter activity.

## Discussion

The major goals of this study were: 1) to determine whether Spry1 and Spry4 play a role on VSMC phenotypic modulation, and 2) to identify mechanisms by which Spry1 and Spry4 regulate VSMC phenotype. We used shRNA knockdown strategies to determine the consequences of loss-of-function of either Spry1 or Spry4, and an adenoviral expression system to overexpress Spry1 or Spry4 in hAoSMC. Together, these two approaches show the novel findings that Spry1 and Spry4 have opposing functions in the regulation of VSMC phenotype. In elucidating the differential functions of Spry1 and Spry4 in VSMC we show that Spry1 increases, while Spry4 decreases *Mycocd* expression in association with regulation of Akt activation. Furthermore, we show that FoxO proteins especially FoxO3a binds to the *Myocd* promoter and represses its expression. Lastly, we show that Spry1 and Spry4 are expressed in the vessel wall of both mouse and humans, suggesting a role for these proteins in vessel wall function.

Spry proteins are feedback inhibitors of RTK signaling and restrain RTK signaling outputs in multiple cell types [11], [12]. Studies indicate that Sprys inhibit RTK-mediated ERK activation by inhibiting Raf1 activation directly or upstream or parallel to Ras [22], [23], [24]. Spry proteins share several structural motifs in common including a C-terminal cysteine-rich domain, a Raf-1 binding domain and a highly conserved N-terminal residue thought to be important to their function. Based upon their structural similarities Sprys are thought to have similar functions in inhibiting downstream RTK signaling. However, here we show that Spry1 knockdown unexpectedly resulted in decreased phosphorylation of ERK and Akt and a decrease in smooth muscle marker gene expression and cell proliferation. This is surprising because loss of Spry1 function in several other contexts results in enhanced signaling downstream of RTKs [25], [26]. However, previous studies have also shown that Spry1 and Spry2 can interfere with ubiquitination and degradation of FGFR1 [27] and EGFR [28], [29], which leads to enhanced RTK signaling. By binding to c-Cbl, Spry2 prevents c-Cbl mediated ubiquitination and degradation of EGFR. Thus Spry1 and Spry2 expression potentiate FGFR and EGFR signaling by increasing their stability. In this context, we have noticed knockdown of Spry1 decreases, while overexpression of Spry1 increases EGFR protein levels (data not shown). Thus it is possible that the decreased Akt and ERK signaling and cell proliferation by loss of Spry1 function in VSMC may result from the decreased levels of EGFR and subsequent downstream signaling. It is also possible that the observed increase in Spry4 expression due to Spry1 knockdown may also account for the decrease in ERK and Akt activation. Detailed studies are ongoing to determine the mechanism of Akt activation by Spry1.

In contrast to the effects of knockdown of Spry1, knockdown of Spry4 resulted in increased ERK and Akt activation with a concomitant increase in the expression of VSMC marker genes and increased proliferation. It is interesting to note that knockdown of Spry4 did not result in a compensatory increase in Spry1 expression. These data suggest that Spry4 has a unique function in the modulation of ERK and Akt signaling in VSMC that cannot be compensated for by expression of Spry1 or Spry2. Although knockdown of Spry2 in VSMC has not been reported, and we have not addressed the loss of Spry2 function in the present study, over expression of Spry2 in VSMC inhibits VSMC proliferation *in vitro*. The apparent non-redundant function of Spry4 in VMSC may be due to a mutual requirement of Spry4 for the function of Spry1 or Spry2. In support of this notion is the observation that Spry1, Spry2, Spry3, and Spry4 can form homodimers and heterodimers, and that these dimers differentially bind to different components of the Ras-Raf-ERK pathway. Indeed it was show that Spry1 and Spry4 heterodimers were the most potent at inhibiting ERK activation [30]. Although Spry1 and Spry4 heterodimers potently inhibited FGF2 mediated ERK activation, it had little effect on EGF mediated ERK activation. Thus it is possible that heterodimers and homodimers have different functions, or function in a cell type-specific manner. Future studies in which all Spry family members are knocked down either individually or in combination will be required to determine the extent to which Spry4 regulation of ERK and Akt in VSMC is dependent upon other Spry family members.

Our overexpression studies also demonstrate unique, non-overlapping functions of Spry1 and Spry4 in VSMC. Overexpression of Spry1 did not have a significant effect on the phosphorylation state of ERK, however, surprisingly, it resulted in a small but statistically significant increase in Akt phosphorylation which was accompanied by increased expression of VSMC marker genes, such as ACTA2, SM22α and CCN1, increased proliferation and migration. In contrast, overexpression of Spry4 in hAoSMC resulted in inhibition of ERK and Akt phosphorylation, expression of contractile genes, proliferation and migration. It is likely that over-expression of either Spry1 or Spry4 favors homodimerization of each of the overexpressed Spry, thus the observed effects are likely due to functional homodimers of the expressed proteins. Because the effects of overexpression of Spry1 and Spry4 are opposite that of the effects of knockdown of these proteins, the data support the concept that balanced expression of these two proteins may be necessary for VSMC homeostasis and that a disturbance in the relative balance of the expression of Spry1 or Spry4 may contribute to aberrant RTK signaling, proliferation and gene expression. In addition, our results show VSMC cell proliferation and differentiation are not necessarily mutually exclusive, that is, termination of proliferation is not necessary for an increase in VSMC marker gene expression [2]. Indeed, during embryonic and postnatal development, VSMC have a high rate of proliferation but also express high levels of VSMC marker genes [31], [32]. In addition, in advanced atherosclerotic plaques, VSMC proliferation is low but there is also significant down-regulation of VSMC contractile genes [33]. Further study is needed to determine how Spry1 and Spry4 integrate hAoSMC cell proliferation and differentiation.

In this report, hAoSMC were cultured in 5% FBS and growth factor enriched SmGM-2 medium, which maintains SMC marker gene expression and VSMC proliferation at same time. The roles of Spry1 and Spry4 on VSMC phenotypic modulation under these conditions mimics more closely the in vivo milieu than stimulation with individual growth factors and warrants further study in vivo. We have observed distinct regulation of Spry1 and Spry4 expression in hAoSMC by stimulation with different growth factors. For example, FGF2 and PDGF-BB down-regulate Spry1, but up-regulate Spry4 expression, while EGF up-regulates both Spry1 and Spry4 expression (data not shown). Indeed, we show that Spry1 and Spry4 are expressed in human atherosclerotic plaques with expression decreased in the neointima. Due to the complexity of the signaling pathways active in atherosclerotic lesions, further study will be required to ascertain under what conditions Spry1 and Spry4 are down regulated or up regulated in atherosclerotic plaques. These observations support the concept that Spry1 and Spry4 play distinct roles in VSMC phenotype modulation. Studies are ongoing to define the roles of Spry1 and Spry4 on individual growth factor mediated signaling pathways in VSMC.

It has been shown that blocking ERK signaling in mouse bone marrow mesenchymal stem cells cultured in low serum condition induces SMC differentiation through up-regulation of myocardin, although the mechanism is not defined [34]. We found that chemical inhibition of ERK in hAoSMC resulted in an increase in VSMC marker gene expression whereas inhibition of Akt decreased marker gene expression. Surprisingly, co-treatment with inhibitors of ERK and Akt resulted in a partial rescue of Akt phosphorylation and less inhibition of marker gene expression than with Akt inhibitor alone. In Spry1 expressing hAoSMC, the effect of the ERK inhibitor was additive with Spry1 at increasing VSMC maker gene expression, whereas the chemical inhibition of Akt decreased Spry1-induced VSMC marker gene expression. These data suggest that Spry1-mediated VSMC marker gene expression is driven principally through the Akt pathway and that ERK signaling acts as a repressor of Akt signaling and downstream gene expression in this context. As mentioned previously, it is possible that overexpression of Spry1 is functioning to enhance EGFR signaling and thus Akt signaling. Further study will be required to determine more precisely how Spry1 enhances Akt signaling.

Although the downstream target genes of Myocd have been described in detail, little is know about the regulation of *Myocd* expression. FoxO3a negatively regulates *Myocd* expression via a catalase/ROS pathway [20]. Here we show an additional mechanism of FoxO-mediated regulation of *Myocd* transcription, in which FoxO, especially FoxO3a, directly binds to the human *Myocd* promoter proximal region to repress its transcription. Knockdown of FoxO proteins increases *Myocd* mRNA levels, supporting a repressive function of FoxO proteins on *Myocd* gene expression in hAoSMC. Our results reveal a novel Spry/Akt/FoxO pathway where increased levels of Spry1 or decreased levels of Spry4 promote Akt activation, phosphorylation of FoxO proteins and the subsequent derepression of the *Myocd* promoter resulting in increased *Myocd* transcription. Analysis of a distal (-20 to -30kb) element upstream of the *Myocd* translational start site showed that *Myocd* is a direct target of Tead, Mef2 and FoxO proteins in mouse cardiogenesis [35]. FoxO, Mef2C and Tead positively regulate this *Myocd* distal enhancer. The definitive role of FoxO proteins in regulating *Myocd* in VSMC requires additional study.

**Figure 6:**
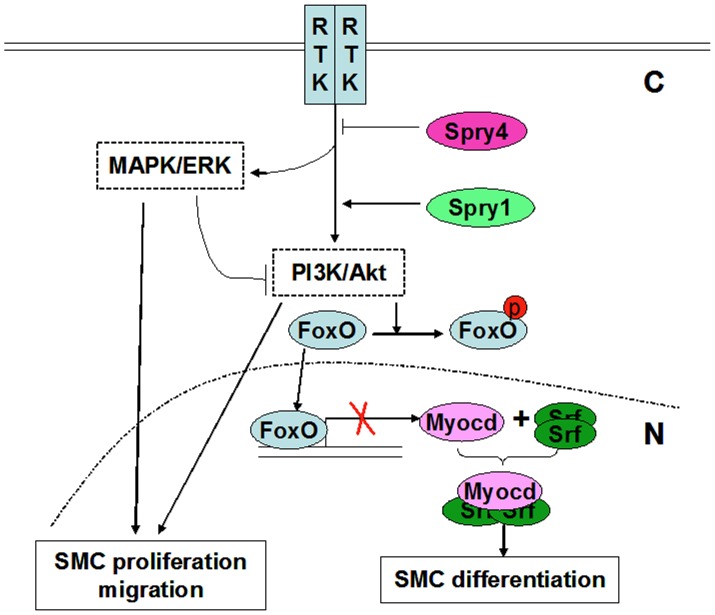
Working model whereby Spry1 and Spry4 have opposing functions in VSMC phenotypic modulation. Spry1 and Spry4 differentially regulate VSMC phenotypes in part by modulating different RTK signaling outputs, where Spry4 inhibits both MAPK/ERK and PI3K/Akt activation and therefore the VSMC proliferation and differentiation, while Spry1 increases PI3K/Akt activation and SMC markers expression. In this model, under conditions of Spry4 excess, Akt activation is low allowing FoxO proteins to bind the *Myocd* proximal promoter and repressing *Myocd* transcription and subsequent VSMC contractile gene expression. Under conditions of Spry1 excess, PI3K/Akt signaling is enhanced leading to increased phosphorylation of FoxO proteins and inhibition of FoxO translocation into the nucleus, hence releasing the repressive functions of FoxO proteins on Myocd and SMC markers expression. Note: C: cytoplasmic, N: nucleus.

In summary, Spry1 and Spry4 play distinct roles in regulating hAoSMC phenotypic modulation through differentially regulating the Akt pathway (Fig. 6). Spry1 promotes hAoSMC differentiation phenotype most likely through increasing Akt activation and subsequently relieving the repressive function of FoxO proteins on *Myocd* transcription, whereas Spry4 inhibits both Akt and ERK activation, VSMC proliferation, migration and differentiation. Hence, the relative balance of Spry1 and Spry4 expression influences whether VSMC exhibit a contractile or synthetic phenotype. Thus, regulating the balance of Spry1 and Spry4 by pharmacological means may provide new avenues for the treatment of vascular disease.

## Supporting Information

Figure S1 **The expression of FoxO isoforms in hAoSMC and their roles in regulation of Myocd transcriptional activity.** A) Immunoblot to show FoxO proteins expression in hAoSMC. B) RT-qPCR to show relative levels of FoxO isoforms. C) Agarose gel to show the relative levels of FoxO isoforms. D) Rat SMA promoter luciferase assay to show that FoxO4 but not FoxO1 and FoxO3 inhibit Myocd induced rSMA (rACTA2) promoter activity. E) Immunoblot to show that FoxO4 but not FoxO3 WT or FoxO3TM inhibit Myocd induced CNN1 expression. F) Inhibition of PI3K/Akt signaling by Ly294002 decreases ectopic expression of Myocd-induced CNN1 expression.
(TIF)

Figure S2 **Spry1 and Spry4 differently regulate Akt/FoxO signal in hAoSMC.** A) Immunofluorescence staining to show that over-expression of Spry1 increases SMA expression in hAoSMC. B) FoxO3a immunofluorescence staining to show that forced expression of Spry4 increases FoxO3 nuclear localization (white arrows). B) FHRE-luciferase reporter assay to show that Spry1 decreases FHRE-luciferase activity; PI3K inhibitor Ly294002 increases FHRE-luciferase activity as control. C) FHRE-luciferase reporter assay to show that inhibition of PI3K by Ly294002 abolishes Spry1 decreased FHRE-luciferase activity.
(TIF)

Table S1 **RT-qPCR primers.**
(DOC)
