# Supplementary figures and images for: Spry1 and Spry4 Differentially Regulate Human Aortic Smooth Muscle Cell Phenotype via Akt/FoxO/Myocardin Signaling
Source: PLoS One. 2013 Mar 15;8(3):e58746. doi: 10.1371/journal.pone.0058746 (PMC3598808; doi:10.1371/journal.pone.0058746)

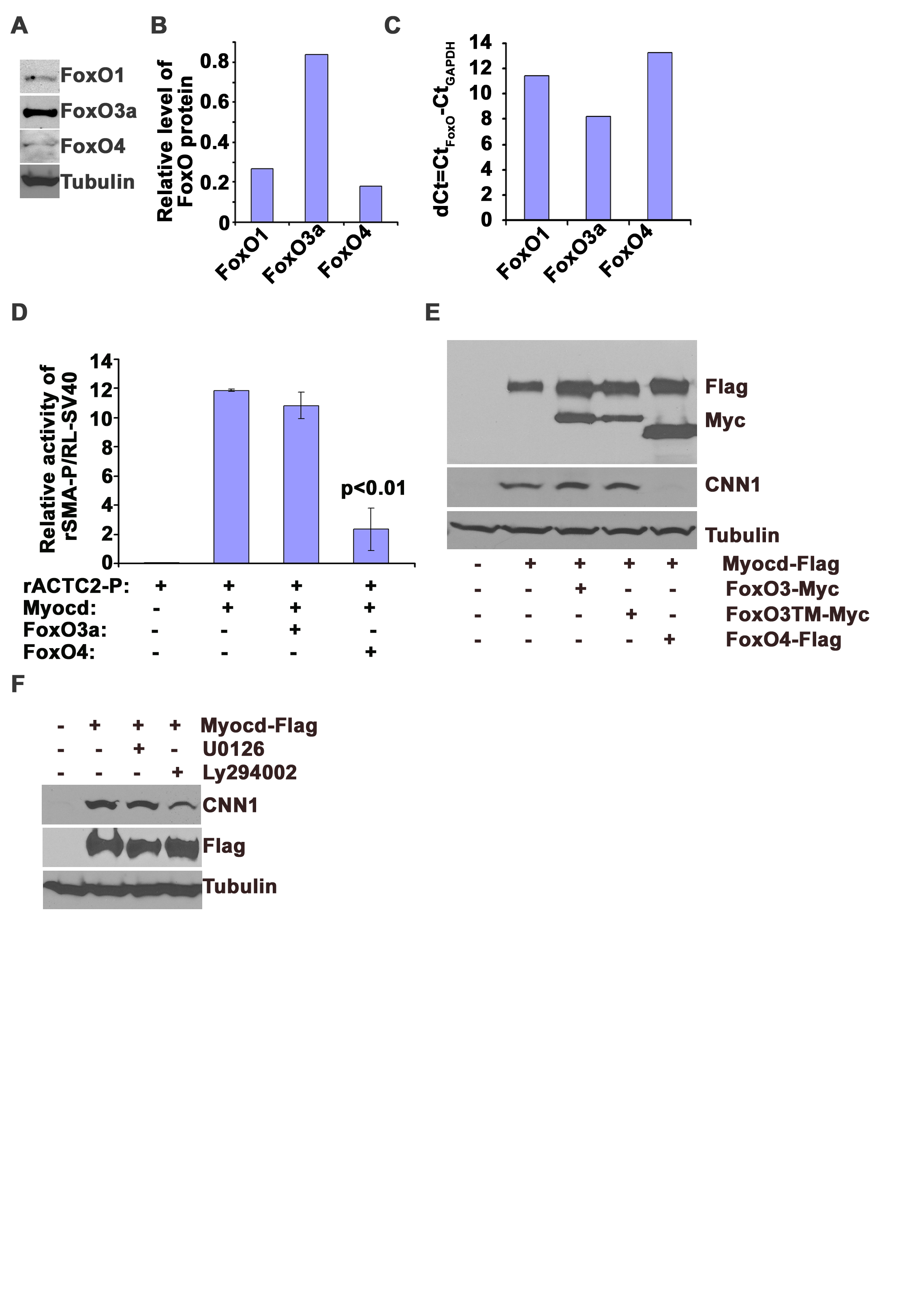

Supplement: Figure S1 — The expression of FoxO isoforms in hAoSMC and their roles in regulation of Myocd transcriptional activity. A) Immunoblot to show FoxO proteins expression in hAoSMC. B) RT-qPCR to show relative levels of FoxO isoforms. C) Agarose gel to show the relative levels of FoxO isoforms. D) Rat SMA promoter luciferase assay to show that FoxO4 but not FoxO1 and FoxO3 inhibit Myocd induced rSMA (rACTA2) promoter activity. E) Immunoblot to show that FoxO4 but not FoxO3 WT or FoxO3TM inhibit Myocd induced CNN1 expression. F) Inhibition of PI3K/Akt signaling by Ly294002 decreases ectopic expression of Myocd-induced CNN1 expression. (TIF) [file pone.0058746.s001.tif]

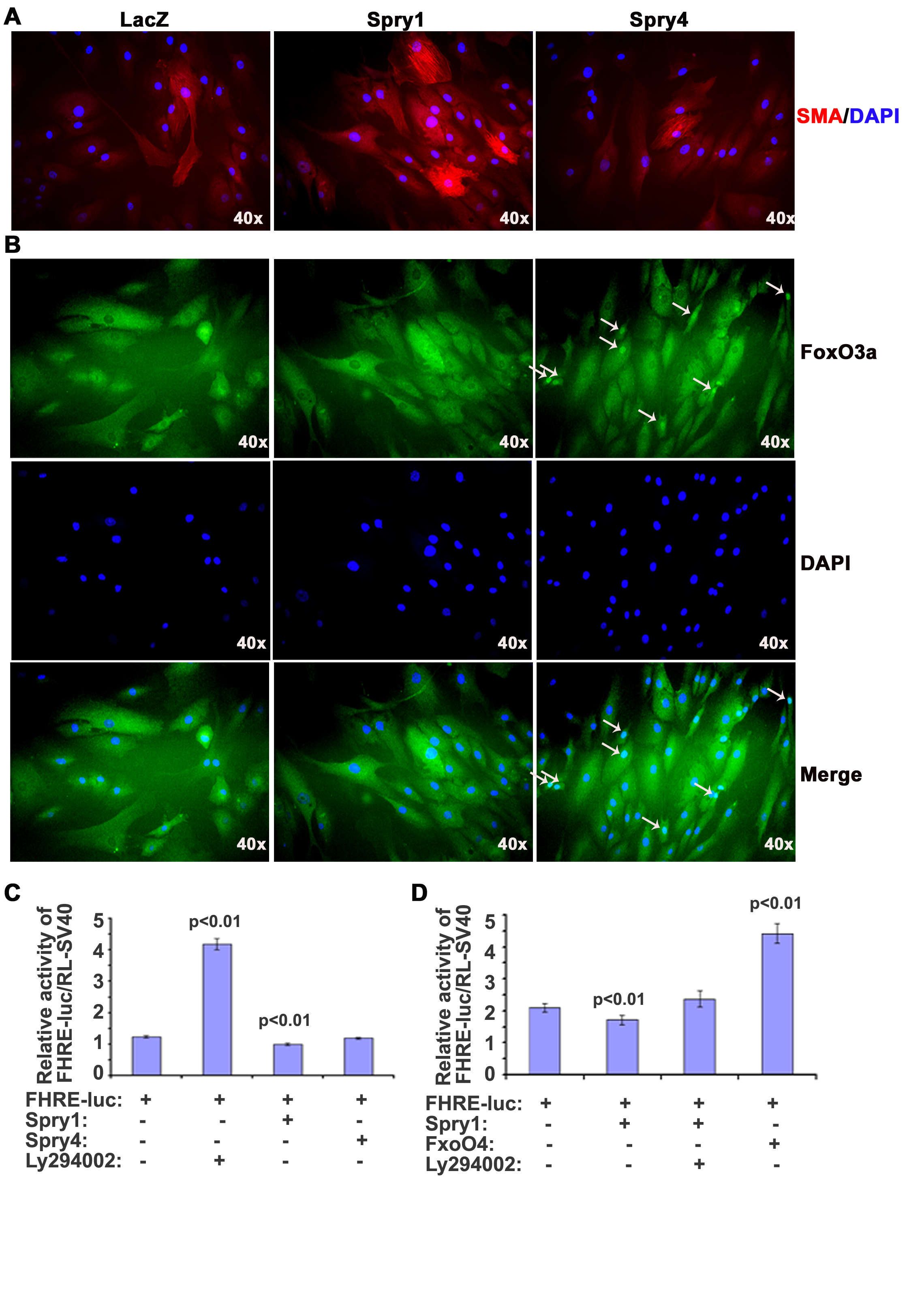

Supplement: Figure S2 — Spry1 and Spry4 differently regulate Akt/FoxO signal in hAoSMC. A) Immunofluorescence staining to show that over-expression of Spry1 increases SMA expression in hAoSMC. B) FoxO3a immunofluorescence staining to show that forced expression of Spry4 increases FoxO3 nuclear localization (white arrows). B) FHRE-luciferase reporter assay to show that Spry1 decreases FHRE-luciferase activity; PI3K inhibitor Ly294002 increases FHRE-luciferase activity as control. C) FHRE-luciferase reporter assay to show that inhibition of PI3K by Ly294002 abolishes Spry1 decreased FHRE-luciferase activity. (TIF) [file pone.0058746.s002.tif]
