# Supplementary material for: Spry1 and Spry4 Differentially Regulate Human Aortic Smooth Muscle Cell Phenotype via Akt/FoxO/Myocardin Signaling
Source: PLoS One. 2013 Mar 15;8(3):e58746. doi: 10.1371/journal.pone.0058746 (PMC3598808; doi:10.1371/journal.pone.0058746)
Supplement: Table S1 — RT-qPCR primers. (DOC) [file pone.0058746.s003.doc]

Table S1. RT-qPCR primers

| Gene name | Primer sequences | Gene bank number |
| --- | --- | --- |
| SM22α | Sense: 5’-AACAGCCTGTACCCTGATGG-3’  Antisense: 5’-CGGTAGTGCCCATCATTCTT-3’ | NM_0111001522.1 |
| ACTC2 | Sense: 5’-AGCCAAGCACTGTCAGGA-3’  Antisense: 5’-ACAATGGATGGGAAAACAG-3’ | NM_001613.2 |
| SM-MHC | Sense: 5’-GCTGGAAGACACACTGGACA-3’  Antisens: 5’-CCAGGTCTGCGTTCTCTTTC-3’ | NM_002474.2 |
| CNN1 | Sense: 5’-AGGCTCCGTGAAGAAGATCA-3’  Antisense: 5’-CCACGTTCACCTTGTTTCCT-3’ | NM_001299.4 |
| GAPDH | Sense: 5’-GAAGGTGAAGGTCGGAGTC-3’  Antisens: 5’-GAAGATGGTGATGGGATTTC-3’ | NM_002046.3 |
| Spry4 | Sense: 5’-AGAAGTGTACTGAAGGGACTGGAG-3’  Antisense: 5’-GTGTGTAGACCACCAAGATCACC-3’ | NM-030964 |
| Spry1 | Sense: 5’-CACTGCTGCTCTAGATACCTGTGT-3’  Antisense: 5’-GCAGCTCTCCAGCTTACAATAGAC-3’ | NM_199327 |
| Myocd | Sense: 5’-TGCATGCTGCTGTAAAGTCC-3’  Antisense: 5’-TAGCTGAATCGGTGTTGCTG-3’ | NM_001146313 |
| FoxO1 | Sense: 5’-AAGAGCTGCCCTACTTCAA-3’  Antisense: 5’-CTGTTGTTGTCCATGGATGC-3’ | NM_002015.3 |
| FoxO3a | Sense: 5’-AGCTGCTTTGGGAGTGAGAA-3’  Antisense: 5’-CCCTTCCTCAGCTGTTTCAG-3’ | NM_001455.3 |
| FoxO4 | Sense: 5’-CTTTGAGCCAGATCCCTGAG-3’  Antisense: 5’-CACCTGTGTGTGACCAGACC-3’ | NM_005938.3 |
